# Downregulation of ERK signaling impairs U2OS osteosarcoma cell migration in collagen matrix by suppressing MMP9 production

**DOI:** 10.3892/ol.2013.1655

**Published:** 2013-11-04

**Authors:** BARUN POUDEL, DO-KUK KIM, HYEON-HUI KI, YOUNG-BAE KWON, YOUNG-MI LEE, DAE-KI KIM

**Affiliations:** 1 Department of Immunology and Institute of Medical Sciences, Chonbuk National University Medical School, Jeonju, Jeonbuk 561-756, Republic of Korea; 2 Department of Oriental Pharmacy, College of Pharmacy, Wonkwang University, Iksan 570-749, Republic of Korea; 3 Department of Pharmacology, Chonbuk National University Medical School, Jeonju 561-756, Republic of Korea

**Keywords:** U2OS, osteosarcoma, extracellular signal-regulated kinase, matrix metalloproteinase 9, PD98059

## Abstract

The present study investigated the role of extracellular signal-regulated kinase (ERK) activation in the migratory phenotype of human U2OS osteosarcoma (OS) cells in a collagen matrix. The activation of ERK was inhibited by PD98059, a specific inhibitor of ERK kinase. Additionally, no significant differences were observed in the adhesion and proliferation of the cells with or without PD98059 treatment in collagen-coated dishes. The migratory capacity of the U2OS cells was then examined in non-coated and collagen-coated dishes, and the results depicted that collagen I enhanced the migration of the U2OS cells, the effect of which was significantly blocked by the treatment of the cells with PD98059. Furthermore, enhanced gene and protein expression of matrix metalloproteinase 9 (MMP9), but not MMP2, was observed to be involved in the enhanced migratory phenotype of the U20S cells in the collagen-coated plates. This effect was partially abolished by the treatment of the cells in the collagen-coated dishes with ERK inhibitor. Collectively, the data demonstrate that ERK signaling is important for the migration of U2OS cells through the extracellular matrix (ECM), which is comprised mostly of collagen, by enhancing MMP9 production. These results may contribute to the regulation of MMP9 production in metastatic OS.

## Introduction

Osteosarcoma (OS) is a highly malignant bone tumor that affects children and adolescents. The availability of neo-adjuvant chemotherapy and surgery have significantly increased the five-year survival rate of patients. However, patients with metastasis, particularly in the lung, show poor survival rates (1). Therefore, elucidation of the molecular events underlying the invasiveness of OS may aid in identifying the new targets for an improved diagnosis and treatment of patients with metastatic OS.

Metastasis of a tumor involves several processes, including increased proliferation of cells, remodeling of tissues and invasion (2). Consistently, cell invasion and migration are carried out by matrix metalloproteinases (MMPs) (3). Most significantly, MMP2 and MMP9 have been reported to cause invasion and metastasis in various cancers (4,5).

MMPs are zinc-dependent endopeptidases whose expression is regulated by proteolytic activation and by selective inhibitory proteins. The majority of the extracellular matrix (ECM) components are the substrates of MMPs (1). Furthermore, MMPs have been reported to process several bioactive factors, apoptotic chemokines and cell signaling factors, which affect immune responses (8). Collagen I is the major ECM component that contributes to the structural and mechanical function of bone (6). MMPs have the capacity to degrade collagen and enhance metastasis and invasion (7). A higher expression of MMPs in malignant tissues compared with normal tissues has been implicated in malignant tumors of the prostate, lung, colon and pancreas, and has been correlated with poor survival rates in patients with such diseases (7).

Extracellular signal-regulated kinase (ERK)-5 belongs to the effector kinase of a mitogen-activated protein kinase (MAPK) signaling pathway. ERK5 has been known to regulate the expression of MMP2 and MMP9 (9,10) and the degradation of the ECM (10). Furthermore, ERK knockdown has been reported to reduce cellular migration and invasion in PC3 cells (10). These studies indicate that ERK may have a major role in cancer cell migration and invasion.

The present study aimed to elucidate the role of collagen in OS by examining morphological features, cellular attachment, proliferation status, expression of MMP2 and MMP9 and ERK-mediated function in migration and invasiveness in an OS cell line.

## Materials and methods

### Cell culture

The human OS U2OS cell line was obtained from the American Type Culture Collection (ATCC, Manassas, VA, USA) and cultured in Dulbecco’s modified Eagle’s medium (DMEM; Cambrex Bio Science Walkersville, Inc., Walkersville, MD, USA) containing 10% fetal bovine serum (FBS; HyClone, Logan, UT, USA) and 1X penicillin-streptomycin in a humidified incubator at 37°C and 5% CO_2_. When confluent, the detachment of cells was performed using 0.25% trypsin and 0.05% EDTA (trypsin-EDTA) for 5–10 min and subcultured at the ratio of 1:5 every three days.

### Morphology

The cells were cultured (2×10^5^ cells/ml) on non-coated or collagen-coated dishes. Following 48 h, the cells were analyzed on a light microscope. Subsequently, the cells that were treated with PD98059 (Bionol, Plymouth Meeting, PA, USA) were also visualized. The comparisons of collagen and/or PD98059-treated cells were performed along with the collagen and/or PD98059-treated or untreated cells.

### Cell attachment assay

The U2OS cells (6×10^4^) were cultured on non-coated or collagen-coated 6-well plates with or without PD98059 for the indicated time-points. Following the adhesion time that was specified for the experiment, the supernatant media and the cells were removed. The adherent layers were then washed with phosphate-buffered saline (PBS) three times, and the adherent cells were harvested using trypsin-EDTA, centrifuged at 400 × g for 5 min, resuspended in a complete medium and their cell counts recorded on a Neubauer hemacytometer (Erma Inc., Tokyo, Japan).

### Cell proliferation assay

The cell proliferation assay was performed using a 3-(4,5-dimethylthiazol-2-yl)-2,5-diphenyltetrazolium bromide (MTT) kit (Amresco, Solon, OH, USA). The cells were plated (5×10^4^/well) on the collagen-coated or non-coated 96-well plates. Subsequently, for the ERK inhibition assay, the cells were either left untreated or were treated with PD98059 (20 μM). The cells were incubated for the indicated time-points and 10 μl MTT solution was added to each well. Further incubation was carried out for 4 h. The plates were read on an ELISA reader at 450 nm.

### Reverse-transcription polymerase chain reaction (RT-PCR)

Total RNA was isolated from the cells using TRIzol reagent (Invitrogen, Carlsbad, CA, USA) according to the manufacturer’s instructions. cDNA synthesis was performed using a cDNA synthesis kit (Invitrogen). The primers and cycling conditions have been previously described elsewhere (11). The PCR products were run on a 1.2% agarose gel and their images were captured.

### Western blotting

To detect the protein expression of phosphorylated or total ERK, MMP2 and MMP9, the cells were detached using 0.25% trypsin-EDTA and washed with PBS. Lysis buffer (Intron, Sungnam, Korea) was used to prepare the total cell lysates. The lysates were electrophoresed using 8% SDS-PAGE and transferred to polyvinylidene difluoride membranes (Amersham Pharmacia Biotech, Inc., Piscataway, NJ, USA). The membranes were incubated with blocking solution that contained rabbit polyclonal phospho-ERK, rabbit polyclonal ERK (Cell Signaling Technology, Beverly, MA, USA), goat polyclonal MMP2 or goat polyclonal MMP9 (Santa Cruz Biotechnology, Inc., Santa Cruz, CA, USA) antibodies. The membranes were then incubated with horseradish-conjugated secondary antibodies (Amersham Pharmacia Biotech, Inc.). An enhanced chemiluminescence detection kit (Amersham Pharmacia Biotech, Inc.) was used to detect the epitope on the proteins that were recognized by the specific antibodies.

### Cell migration assay

The U2OS cells were plated in collagen-coated or non-coated 12-well plates until confluence in order to study the effect of collagen and ERK on the OS cell migration assay. A linear wound was gently created in the monolayer using a sterile yellow pipette tip, followed by washing with the complete medium to remove the cellular debris in order to yield an acellular line per well. The cells were incubated with ERK inhibitor for 48 h. Images of the wounded areas were captured and the location on the dish was noted in terms of the distance between the cells.

## Results

### Collagen I induces changes in U2OS cells

**Figure 1:**
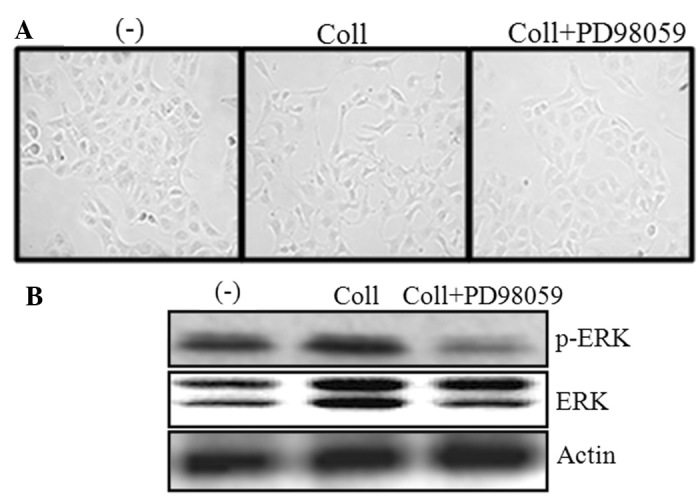
Effect of collagen I and ERK inhibitor on the cellular morphology of U2OS cells. (A) Morphological changes in the untreated control (−), collagen I-treated (coll) and collagen I and ERK inhibitor-treated (Coll+PD98059) cells following 24 h (magnification, ×40). (B) The cells were treated with collagen I and/or PD98059 or left untreated for 24 h, lysed with lysis buffer and analyzed for ERK activity by western blotting. The data represent one of three independent experiments. ERK, extracellular signal-regulated kinase.

The U2OS cells were cultured on collagen-coated or non-coated plates. Images were captured following 48 h using a 40X objective lens. Fig. 1A shows that the cells underwent morphological changes that were similar to the epithelial to mesenchymal transition (EMT) when they were cultured on the collagen-coated plate. The prominent feature was the scattering of the cells that were plated on the collagen-coated dishes. However, there was no significant difference in the cellular morphology between the PD98059-treated or untreated cells in the collagen-coated dishes. Furthermore, the effect of PD98059 on the phosphorylation of ERK was investigated. The data revealed that PD98059 significantly inhibited the phosphorylation of ERK (Fig. 1B).

### Proliferation of U2OS is enhanced by collagen I

**Figure 2:**
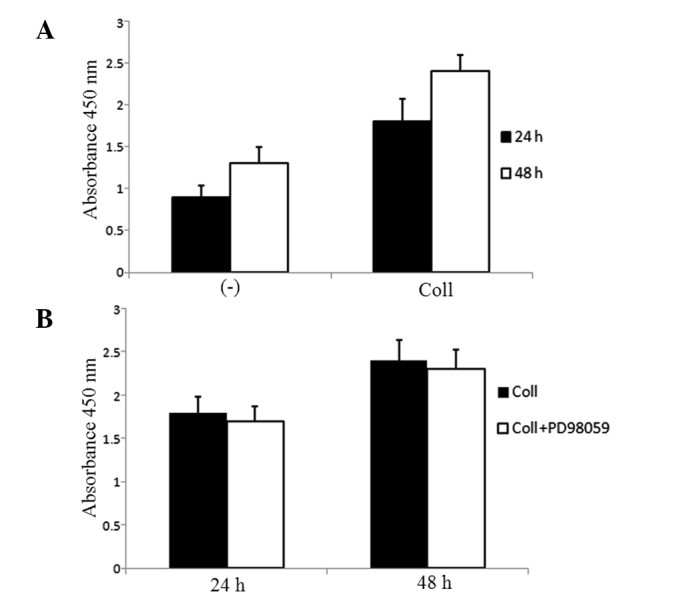
Effect of collagen I and ERK inhibitor treatment on the proliferation of U2OS cells. (A) A proliferation assay was performed using collagen I and the untreated control for the indicated time-points. (B) The cells on the collagen-coated dishes were treated with ERK inhibitor and compared with the collagen-coated dishes cells for the indicated time-points. The data represent the mean of three independent experiments. ERK, extracellular signal-regulated kinase; (−), control; coll, collagen I-treated cells; Coll+PD98059, collagen I and ERK inhibitor-treated cells.

An evaluation of the effect of collagen I on the proliferation of the U2OS cells in the collagen-coated plates was performed. Fig. 2A shows that the U2OS cells proliferated significantly in the collagen-coated dishes compared with the negative control. In order to elucidate the effect of ERK inhibition on cellular proliferation, ERK expression was inhibited in the cells using PD98059. However, there was no significant difference in the proliferation of the U2OS cells between the untreated or treated cells (Fig. 2B).

### Adhesion of U2OS cells

**Figure 3:**
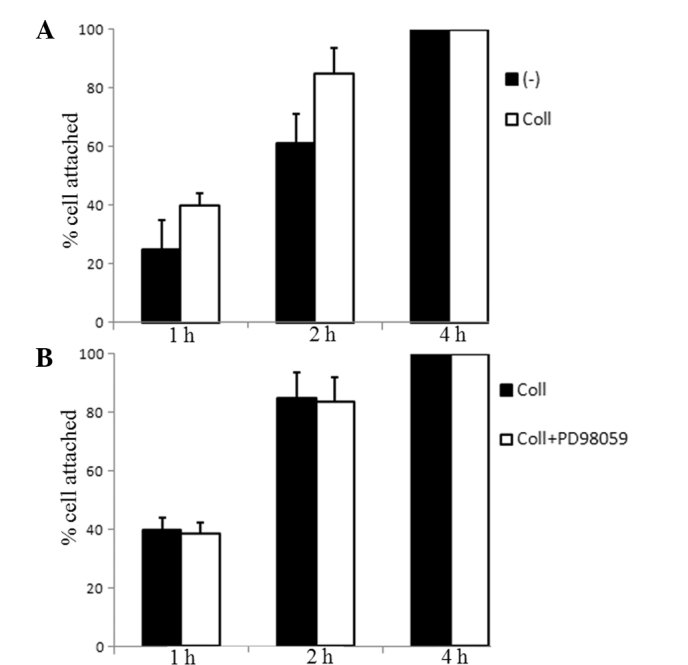
Effect of collagen I and ERK inhibitor on the adhesion of U2OS cells. (A) The adhesion profile of the U2OS cells upon collagen treatment compared with the non-treated control was performed as described in the Materials and methods section. (B) The effect of ERK inhibition on the collagen-treated cells. The data represent the mean of three independent experiments. ERK, extracellular signal-regulated kinase; (−), control; coll, collagen I-treated cells; Coll+PD98059, collagen I and ERK inhibitor-treated cells.

The cell numbers that adhered to the collagen-coated plates compared with the non-coated plates were determined within the indicated time-points. Fig. 3A shows that compared with the negative control, the U20S cell numbers that adhered to the collagen I-coated plates was higher. When the cells were treated with PD98059 and cultured on collagen-coated dishes for up to 4 h, there was no significant effect on the adhesion of the U2OS cells (Fig. 3B).

### ERK inhibition suppresses the migratory phenotype of U2OS cells

**Figure 4:**
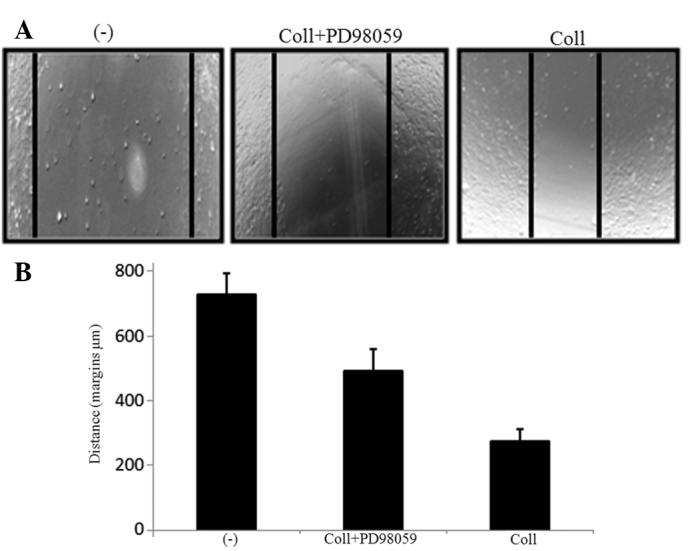
Effect of collagen I and ERK inhibition on the migration of U2OS cells. (A) The migratory capacity of the U2OS cells in the non-treated plates was compared with the collagen I-treated culture plates and with the collagen and ERK inhibitor-treated cells using a wound healing assay (magnifcation, ×10). (B) The distance between the migrated cell margins was noted for three independent experiments and represented in a bar diagram. ERK, extracellular signal-regulated kinase; (−), control; coll, collagen I-treated cells; Coll+PD98059, collagen I and ERK inhibitor-treated cells.

A wound healing assay was performed in order to examine whether ERK inhibition played a role in the invasive characteristics of the U2OS cells. The U2OS cells were treated with or without ERK inhibitor on the collagen-coated dishes. Following 48 h, the migration of the cells along the scratched section was measured. The number of cells that migrated along the wound area was significantly higher in the collagen-coated dishes than in the non-coated dishes (Fig. 4). When compared with the PD98059-treated or untreated cells on the collagen-coated dishes, it was observed that ERK inhibition significantly suppressed the invasion of the U2OS cells in the scratch area. These results illustrate that ERK pathway inhibition suppresses the migratory capacities of U2OS cells.

### ERK inhibition suppresses MMP9 expression

**Figure 5:**
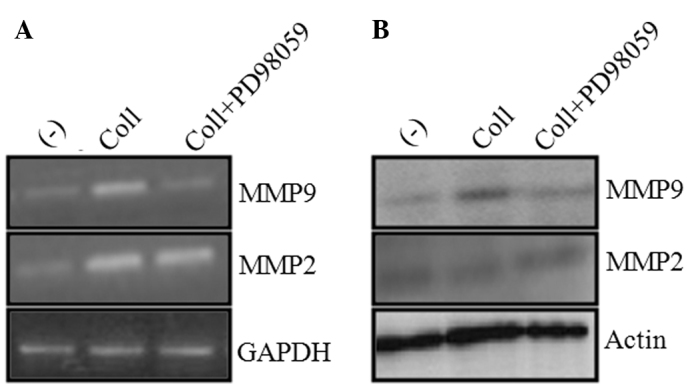
Effect of collagen I and ERK inhibition on MMP2 and MMP9 mRNA and protein expression. (A) The mRNA expression of MMP2 and MMP9 and (B) their protein expression was analyzed as described in the Materials and methods section. The data represent one of three independent experiments. ERK, extracellular signal-regulated kinase; MMP, matrix metalloproteinase; (−), control; coll, collagen I-treated cells; Coll+PD98059, collagen I and ERK inhibitor-treated cells.

MMP2 and MMP9 expression was investigated in U2OS cells in the presence or absence of collagen and/or ERK inhibitor. MMP2 and MMP9 were selected as they have previously been implicated in the invasive ability of cancer cells (11). Upon ERK inhibition, the transcriptional activities of collagen-induced MMP9 was markedly reduced. However, there was no reduction in MMP2 expression between the PD98059-treated or untreated cells in the collagen-coated dishes (Fig. 5A). Consistent with these results, the protein expression of MMP9 was suppressed in the ERK inhibitor-treated cells in the collagen-coated dishes. In addition, no significant difference in MMP2 protein expression was observed between the ERK inhibitor-treated and untreated cells on the collagen-coated dishes (Fig. 5B). These results indicate that MMP9 is the target of ERK signaling in U2OS cells. Further studies are required to elucidate the role of MMP9 in the ERK-related invasive characteristics of U2OS cells.

## Discussion

The present study investigated the role of collagen I in the morphology, adhesion, proliferation and invasive ability of U2OS cells and the response of the ERK pathway in collagen-treated cells. Collagen I was observed to cause the EMT-like phenotype in the U2OS cells. In comparison with the cells in the non-coated dishes, the cells in the collagen-coated dishes demonstrated a scattering behavior, which is consistent with the EMT-like phenotype (12). Consistent with previously reported data (13), the present study observed that collagen I upregulated the proliferation of the U2OS cells. Treatment with PD98059 downregulated collagen-induced MMP9 expression and subsequently, the invasive phenotype of the U2OS cells on the collagen-coated plates was diminished. However, no significant differences in the adherence and proliferation of the U2OS cells were observed between the collagen I-treated and PD98059-treated cells in the collagen-coated plates.

Enhanced production of MMP9 correlates to the invasive phenotype of cancer cells (14,15). One study has reported that MMP9 overexpression in prostate cancer is associated with ERK overexpression (9). The present study aimed to examine the effect of MMP9 and ERK signaling on the invasive ability of U2OS cells. Consistent with a previous study (9), it was observed that ERK upregulated the expression of MMP9 and caused the upregulation of the invasive capacity of the U2OS cells. Similar to the present results, another previous study demonstrated that ERK silencing using siRNA significantly downregulated MMP9, but not MMP2, in OS cells (16).

The present study revealed that collagen caused an EMT phenotype on the U2OS cells. Furthermore, collagen enhanced the adhesion and proliferation of the U2OS cells. However, ERK-5 inhibition using PD98059 had no significant effect on the adhesion and proliferation of the U2OS cells in the collagen-coated dishes. Furthermore, ERK inhibition downregulated the invasive phenotype of the U2OS cells through a suppressive effect on MMP9 expression. Taken together, the data reveal that ERK may be a potent therapeutic target for OS with invasive characteristics.
